# Targeting metabolic reprogramming to overcome immune tolerance in melanoma immunotherapy

**DOI:** 10.3389/fimmu.2025.1597770

**Published:** 2025-06-05

**Authors:** Qinchen Wang, Lei Shi, Daoming Shi, Ningzheng Tai, Xu Wang

**Affiliations:** ^1^ Department of Burns and Plastic Surgery, Affiliated Hospital of Jiangsu University, Zhenjiang, China; ^2^ Department of Thoracic Oncology, Cancer Institute of Jiangsu University, Affiliated Hospital of Jiangsu University, Zhenjiang, China

**Keywords:** melanoma, immune tolerance, metabolic pathways, tumor microenvironment, gut microbiota, immunotherapy

## Abstract

Significant advances in the treatment of melanoma, the most aggressive form of skin cancer, have been achieved via immunotherapy. Despite these improvements, therapeutic resistance remains a formidable challenge, compromising the treatment efficacy and patient outcomes. This review delves into the intricate mechanisms driving immunotherapy resistance in melanoma, emphasizing alterations in key metabolic pathways, changes within the tumor microenvironment, and the critical role of the gut microbiota. This review also examines how metabolic reprogramming supports tumor proliferation and immune evasion, it highlights the impact of extracellular acidification and angiogenic processes on resistance development. By synthesizing current insights, this review emphasizes the importance of targeting these multifaceted interactions to overcome resistance, thereby paving the way for more effective and durable therapeutic strategies in melanoma treatment.

## 1 Introduction

Melanoma is increasingly being diagnosed, particularly among younger populations. Although immunotherapy has significantly improved patient prognosis, research has indicated that melanoma progression involves multiple metabolic pathways linked to oncogene activation and immune tolerance, so existing immunotherapies cannot be satisfactory for all patients.

Metabolic alterations are linked to the development of melanoma, a prominent feature of which is the “Warburg effect,” in which transformed melanocytes rely predominantly on glycolysis for energy, facilitating rapid growth (1, 2). Even under hypoxic conditions, melanoma cells convert glucose to lactate and utilize oxidative phosphorylation, further promoting tumor progression (3). In addition to glycolysis, amino acid metabolism and lipid metabolism undergo metabolic reprogramming in melanoma cells, enabling energy production, redox balance, and adaptation to acidic microenvironments. These adaptations support tumor proliferation, growth, and immune evasion. Additionally, metabolic alterations in the gut microbiota influence melanoma progression.

However, how the above mechanisms lead to immunosuppression remains unclear. Therefore, clinical treatment strategies can be informed and treatment outcomes can be improved by understanding the interaction between tumors and immune cell metabolism and the mechanisms that generate drug resistance.

## 2 Metabolism and immune tolerance

### 2.1 Glycolytic metabolism and immune tolerance

**Figure 1:**
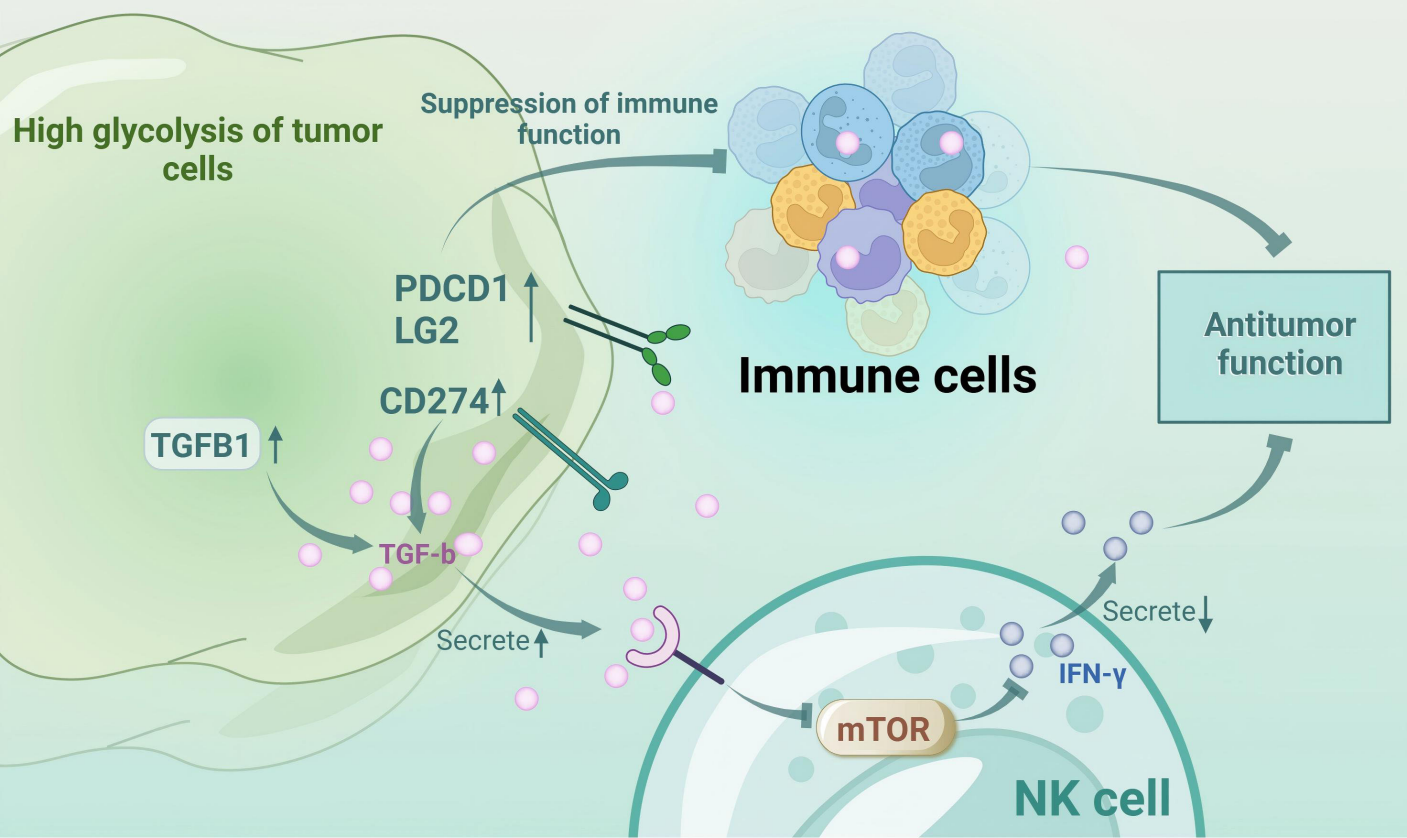
Interactions between tumor metabolism and immune cells in melanoma.

Glycolysis modulates tumor immunology through immune checkpoint regulation, gene expression, and cytokine secretion, particularly in melanoma (4). Enhanced glycolysis leads to the accumulation of lactate, which inhibits approximately 95% of immune cell proliferation, attenuates cytokine secretion and activity, and supports regulatory T cells (Tregs) (5, 6). Monocarboxylate transporter-1 (MCT-1) on cytotoxic T lymphocytes (CTLs) exports lactate; however, excessive lactate impairs the functionality of CTLs despite their increased presence (5). Lactate-induced extracellular acidification via monocarboxylate transporter-4 (MCT-4) inhibits nuclear factor of activated T cells (NFAT) in T and natural killer (NK) cells, reducing interferon-γ (IFN-γ) production and antitumor activity (7). Competition for glycolytic resources with melanoma cells weakens the T cell efficacy and promotes Treg differentiation, facilitating immune evasion (8). Additionally, lactate upregulates vascular endothelial-derived growth factor (VEGF), driving tumor-associated macrophages to the protumorigenic M2 phenotype (9). High-glycolytic tumors express elevated levels of immune inhibitors, such as TGF-B1, CD274, and PDCD1LG2, which suppress immune functions (10). TGF-β inhibits the mammalian target of rapamycin (mTOR) pathway in NK cells, decreasing IFN-γ and antitumor responses (11). Thus, targeting glycolysis may mitigate immune evasion and resistance in melanoma. However, whether the efficacy of immunosuppressants can be improved by directly inhibiting melanoma glycolysis remains to be seen. (**Figure 1** Interactions between tumor metabolism and immune cells in melanoma).

The mitogen-activated protein kinase (MAPK) pathway is intricately linked to glycolysis; the v-raf murine sarcoma viral oncogene homolog B (BRAF)/MAPK pathway is initiated by growth factors, cytokines or hormones binding to a membrane-bound receptor tyrosine kinase (RTK), which induces an interaction between activated RAS and the RAF domain, modulating the shift from oxidative phosphorylation (OXPHOS) to glycolysis in melanoma cells (12, 13). In the MAPK pathway, the BRAFV600E mutation negatively regulates the microphthalmia-associated transcription factor (MITF)-peroxisome proliferator-activated receptor-gamma coactivator 1-alpha (PGC1α) axis, thereby promoting glycolysis (14). BRAF inhibitors (BRAFis) can inhibit the driving effect of hypoxia-induced factor 1α (HIF1α) and Myc on glycolysis, and they can also enhance the tolerance of PGC1α to oxidative stress, which not only maintains the energy balance but also provides a survival environment for tumor cells, thus affecting the therapeutic effect (15). Pyruvate kinase M2 (PKM2) is upregulated in melanoma and resistant cells; its inhibition suppresses growth and resensitizes cells to BRAFis (16). Although combining BRAFis with mitogen-activated protein kinase kinase inhibitors (MEKis) can improve patient prognosis and inhibit the growth of advanced melanoma, this combination cannot achieve an eradication effect. More importantly, upregulation of glycolysis generates resistance to BRAFis and MEKis, allowing previously suppressed melanoma cells to regain their growth advantage, leading to tumor recurrence (17, 18). These mechanisms indicate that glycolysis modulation is pivotal in resistance development.

### 2.2 Amino acid metabolism and immune tolerance

Amino acids, including glutamate, cysteine, leucine, tryptophan, and arginine, play pivotal roles by supporting melanoma proliferation and facilitating antitumor immunity (4). Although amino acid depletion can activate the inositol-requiring enzyme 1α (IRE1α) and retinoic acid-inducible gene 1 (RIG1) pathways to increase cytokine production and the immune response, amino acid depletion impairs essential immune function, making potential strategies for reducing amino acids to enhance antitumor immunity infeasible (19).

#### 2.2.1 Glutamic acid-cysteine and glutamine

In melanoma, PD-1 antibody therapy enhances IFN-γ secretion by CD8^+^ T cells, leading to suppression of the glutamate–cysteine antiporter system Xc⁻ subunit. This suppression induces cysteine depletion and ferroptosis, ultimately compromising immune function (20, 21). Glutamine (Gln) is metabolized to glutamate by glutaminase (GLS), the rate-limiting enzyme in Gln catabolism, which is essential for tumor development (22). In temozolomide (TMZ)-resistant melanoma cells, both Gln metabolism and GLS expression are upregulated, and overexpression of miR-203, which targets GLS, can reverse TMZ resistance (23). The expression of Gln and GLS is greater under long-term action of BRAFis. When melanoma cells are resistant to BRAFis, inhibition of GLS can increase their sensitivity, but the clinical application of GLN as a drug resistance-related factor is not clear (12).

#### 2.2.2 Tryptophan

In melanoma, rat sarcoma (RAS) activation causes tryptophan deficiency, which affects mRNA translation and increases the sensitivity of melanoma cells to immune surveillance (24, 25). However, tryptophan (Trp) depletion concurrently starves cytotoxic T cells and activates immunosuppressive regulatory T cells (Tregs), hindering an effective antitumor response (26, 27).

Dysregulated activation of indoleamine 2,3-dioxygenase (IDO1) and tryptophan 2,3-dioxygenase (TDO) in tryptophan metabolism significantly alters the tumor microenvironment (TME), and simultaneous accumulation of kynurenine (Kyn) activates the aryl hydrocarbon receptor (AhR), promoting the production of FoxP3^+^ regulatory T cells, which eventually leads to immune escape (4). Within the TME, IFN-γ induces excessive activation of IDO1 and TDO in both melanoma cells and tumor-infiltrating lymphocytes, leading to tryptophan depletion, thereby enhancing immune recognition (24). Additionally, an elevated kynurenine-to-tryptophan (Kyn/Trp) ratio in peripheral blood, driven by IDO1 activity, is associated with resistance development and poor prognosis in patients receiving PD-1 antibody therapy (28). Although many studies have focused on the effects of inhibiting the IDO1 pathway, no significant immune efficacy has been achieved. Therefore, further exploration of the impact of tryptophan metabolism on immune effects is needed.

#### 2.2.3 Arginine and branched-chain amino acids

L-arginine plays a pivotal role in immunomodulation. Sufficient levels of L-arginine promote proliferation of T cells and their differentiation into central memory-like T cells, which increases survival rates and enhances antitumor efficacy (29). The immunosuppressive mechanism of myeloid-derived suppressor cells (MDSCs) promotes the expression of arginase (ARG)-1, which depletes the L-arginine required for T cell functional activity, leading to T cell dysfunction and reduced immunotherapy efficacy (30).

Branched-chain amino acid transaminase 1 (BCAT1) expression is markedly elevated in melanoma cells, which mediates the metastasis of specific nitrogen atoms in branched-chain amino acids, and inhibition of BCAT1 suppresses tumor proliferation (31, 32). Leucine (Leu) at elevated levels synergizes with anti-PD-1 antibodies to augment the antitumor activity of immune cells (33). In contrast, leucine deficiency impairs mTORC1 signaling in a RagD-dependent manner, thereby delaying T cell-mediated clearance of melanoma cells. Furthermore, under leucine-depleted conditions, mTOR signaling, which maintains the initial c-Myc expression in NK cells, is disrupted (4). Melanoma cells harboring BRAF mutations exhibit a heightened dependence on leucine; consequently, leucine deficiency may impede autophagy within tumor cells, suggesting a potential novel strategy for immunotherapy (34).

### 2.3 Lipid metabolism and immune tolerance

#### 2.3.1 Lipid metabolism and immune modulation in melanoma

**Figure 2:**
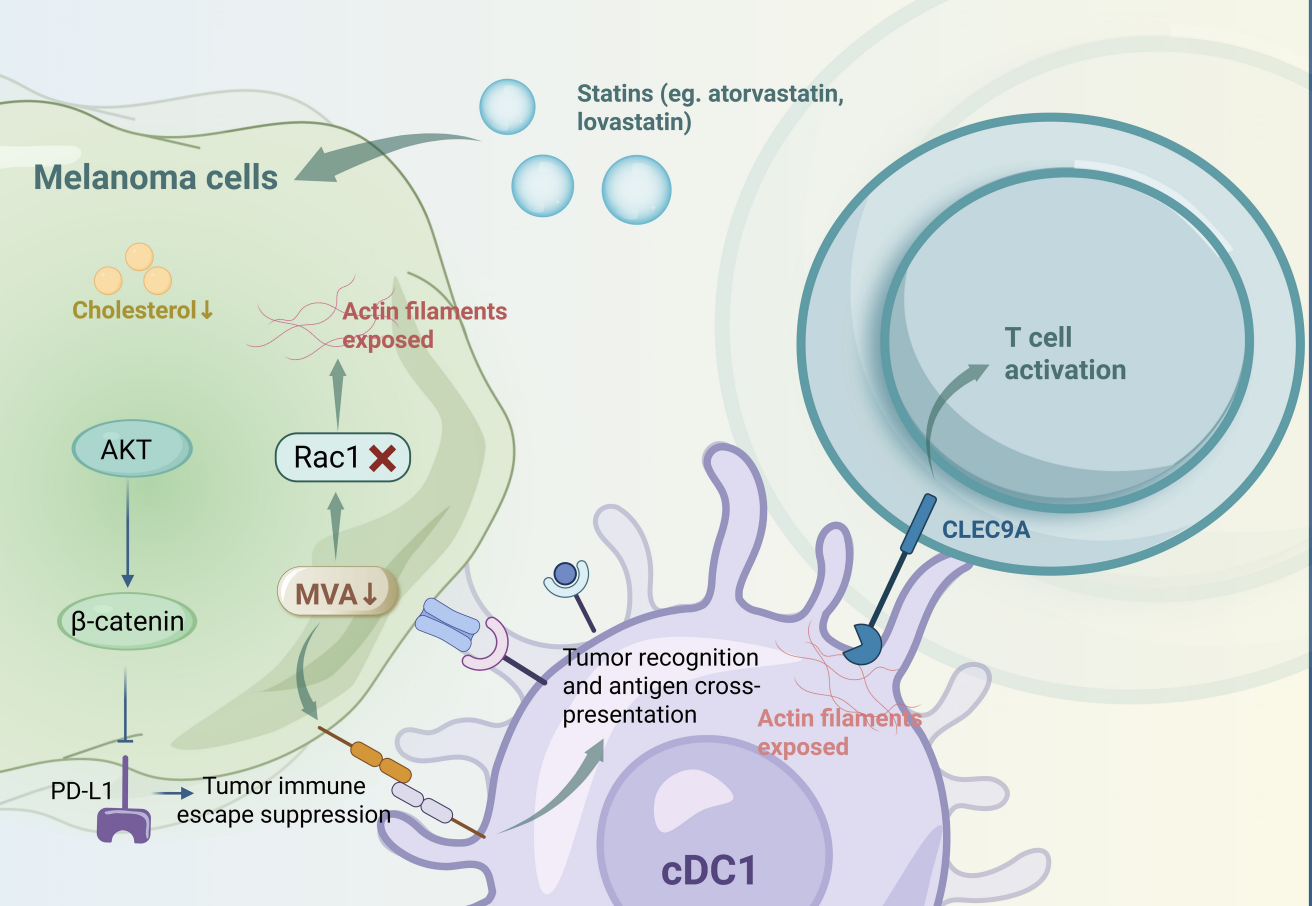
Metabolic reprogramming-mediated immune tolerance in melanoma.

Lipids function as both energy reservoirs and essential structural components for melanoma proliferation, and they undergo substantial modifications during tumor progression (35). These lipid alterations modulate the immunogenicity of melanoma cells and the phenotypes of immune cells, thereby regulating immune evasion and response to immunotherapy, which are crucial for the therapeutic efficacy (4). Statins, such as atorvastatin and lovastatin, inhibit the mevalonate (MVA) and cholesterol biosynthesis pathways, resulting in reduced programmed cell death ligand 1 (PD-L1) expression in melanoma cells via protein kinase B (AKT)- and β-catenin-dependent mechanisms. This downregulation enhances the efficacy of PD-1 antibody therapy in preclinical tumor models (36). Additionally, MVA pathway inhibition in tumor cells promotes antitumor immunity mediated by type 1 conventional dendritic cells (cDC1s) through enhanced tumor recognition and antigen cross-presentation. This inhibition also disrupts Rac family small GTPase 1 (Rac1) prenylation, exposing actin filaments that are recognized by c-type lectin domain family 9 member A (CLEC9A) on cDC1s, thereby activating T cells (37). However, the varying impacts of different lipid metabolic pathways on melanoma cell immunogenicity during lipid remodeling (4). (**Figure 2** Metabolic reprogramming-mediated immune tolerance in melanoma).

#### 2.3.2 Therapeutic implications of lipid pathway modulation

Preclinical studies have confirmed that the antitumor function of CD8+ T cells in melanoma is enhanced by the combined action of fatty acid metabolism and anti-PD-1 antibodies; immunotherapy-induced IFN-γ production suppresses solute carrier family 7 member 11 (SLC7A11) expression, enhances lipid oxidation, and promotes ferroptosis, thereby improving tumor control (20, 38). Additionally, high expression of the CD36 membrane-bound exogenous lipid transporter enables melanoma cells to absorb dietary lipids, promoting metastasis. Inhibition of CD36 expression suppresses melanoma metastasis and improves patient prognosis (39). Moreover, melanoma cells upregulate ATP-citrate lyase (ACLY) and sterol regulatory element-binding proteins (SREBPs) to activate *de novo* lipogenesis. Inhibition of these enzymes results in tumor regression, highlighting the significant impact of lipid alterations on melanoma cell viability (40, 41). The ACLY lipid synthesis enzyme activates the P300 acetyltransferase, leading to histone acetylation at the microphthalmia-associated transcription factor (MITF) locus and increased transcription of the MITF-PGC1α axis, promoting melanoma progression and resistance to MAPK inhibitors (42). Conversely, sterol regulator element binding (SREBP-1) inhibition increases immunotherapy sensitivity, linking lipid synthesis enzyme activation to immunotherapy resistance (43). However, a previous study on uveal melanoma revealed a metabolic shift toward lipid production during tumor growth, which promotes tumor cell growth and increases the metastasis rate (44). Thus, targeting lipid-related pathways is a promising strategy for enhancing immunotherapeutic outcomes in patients with melanoma.

## 3 Microenvironment acidification and immune tolerance

An acidic extracellular milieu profoundly impacts tumor cell proliferation, survival, migration, and invasion, serving as a pivotal hallmark of malignancies (45). In solid tumors, including melanoma, a reversed pH gradient is observed relative to that for normal cells. Specifically, tumor cells maintain an intracellular pH (pHi) that exceeds 7.4, whereas the extracellular pH (pHe) ranges between 6.7 and 7.1, highlighting the distinct pH profiles between malignant and nonmalignant cells (45). Matrix metalloproteinase 2 (MMP2) and interleukin-8 (IL-8) are secreted by melanoma in acidic environments, and they can accelerate the degradation of the extracellular matrix and enhance the invasion and metastasis of tumor cells (46). Most melanoma cells die as they adapt to an acidic environment, but the melanoma cells that survive become more aggressive in a normal environment, leading to treatment resistance (47). In addition, MITF is downregulated in acidic environments and resists the action of MAPKis (48).

Alterations in tumor angiogenesis play critical roles in modulating the extracellular acidity, facilitating the formation of new vascular networks, and increasing the spatial separation between tumor cells and blood vessels, which influence the metabolic preferences of tumor cells, thus dictating a shift between glycolysis and OXPHOS (49, 50). Moreover, short-term interactions between melanoma cells and an acidic microenvironment induce vascular remodeling and impair lymphatic drainage (51). During melanoma progression, overproduction of lymphangiogenic factors alongside diminished levels of lymphangiogenesis inhibitors promotes lymphatic growth at specific stages (52). Vascular endothelial growth factor C (VEGF-C), which is a lymphangiogenic factor secreted by melanoma cells and tumor-associated macrophages (TAMs), is upregulated in the A375P melanoma cell line under acidic conditions, and the expression of VEGF-C depends on the nuclear factor kappa-B (NF-κB) activity (53). Notably, esomeprazole has been demonstrated to inhibit VEGF-C expression in melanoma cells subjected to acidic environments (53).

Carbonic anhydrase IX (CAIX) has emerged as a crucial regulator of pHi in tumor cells (54). CAIX overexpression is correlated with malignancy across various tumor types (55). In metastatic melanoma cells, CAIX expression is significantly elevated when cells are cultured in acidified media, both transiently and chronically (51). Treatment with FC16-670, a CAIX inhibitor, effectively suppresses CAIX expression and induces cell death under acidic conditions, indicating that CAIX activity is indispensable for melanoma cell survival in an extracellularly acidified environment (51). Collectively, these findings underscore the intricate interplay among metabolic pathways, angiogenic processes, and pH regulation in shaping the tumor microenvironment, suggesting potential therapeutic targets for combating malignancy.

## 4 Gut microbiota metabolism and immune tolerance

### 4.1 Dual role of the gut microbiota in tumor immune regulation

In recent years, the gut microbiota has emerged as a pivotal regulator of tumor progression. Empirical studies have revealed that melanoma patients predominantly harboring the *Bacteroides* genus within their gut microbiota tend to exhibit elevated levels of circulating CD4^+^ and CD8^+^ T cells. In contrast, individuals with a gut microbiota dominated by the *Prevotella* genus display increased populations of regulatory Tregs and MDSCs in their peripheral blood (56). Notably, the gut microbiota can negatively regulate MDSCs, thereby diminishing immune evasion and decelerating tumor progression. Additionally, the gut microbiota can activate antigen-presenting cells (APCs), which may inhibit tumor immune escape (57). The gut microbiota plays dual roles in tumor regulation, as antitumor responses within the TME can be attenuated by elevated levels of inflammatory cells and cytokines, thereby facilitating tumor immune evasion. Using a melanoma mouse model, researchers reported that mice subjected to a high-fat diet present increases in the *Clostridia* and *Desulfovibrio* populations. This shift in population activates the HMG-B1/NF-κB signaling pathway in macrophages, leading to secretion of the C-C motif chemokine ligand 2 (CCL2) and tumor necrosis factor-α (TNF-α) chemokines, which in turn promotes MDSC infiltration and tumor metastasis (58).

### 4.2 Gut microbiota as a modulator of the PD-1 antibody therapeutic efficacy

Moreover, the gut microbiota has been identified as a key component of antitumor immunity in the context of PD-1 antibody therapy. Research has indicated that mice colonized with fecal microbiota from melanoma patients who achieved a complete response to PD-1 antibody therapy develop significantly smaller MC38 tumors when treated with anti-PD-L1 monotherapy than mice colonized with fecal microbiota from nonresponding patients (59). Furthermore, the combination of anti-PD-L1 and anti-PD-L2 therapies markedly enhances the antitumor response in mice harboring nonresponder fecal microbiota, resulting in a substantial increase in the overall survival. Additionally, the combined administration of anti-PD-L2 and anti-PD-L1 treatments tends to reduce the growth of ovalbumin-expressing B16 melanoma tumors (B16-L1) (60). These findings underscore the critical role of gut microbiota modulation in melanoma development and the emergence of resistance to immune therapies. However, unlike PD-1 antibody therapy, not all combination therapies have an antitumor effect.

### 4.3 Therapeutic interventions targeting the gut microbiota for melanoma treatment

Accumulating evidence supports the potential of fecal microbiota transplantation (FMT) in mitigating immune tolerance in patients with melanoma. FMT alters the gut microbiota composition of the recipient to enrich beneficial bacteria that regulate tumor immunity and potentially enhance therapeutic outcomes. In melanoma mice transplanted with *Bacteroides*, the expression of chemokines and antigen presentation-related genes increases, which promotes the activation of dendritic cells (DCs) and CD8+ T cells in tumor cells, thereby inhibiting tumor growth (61). In a study conducted at the Hillman Cancer Center at the University of Pittsburgh Medical Center (UPMC) involving 15 participants undergoing FMT, 3 of 5 patients demonstrated positive clinical responses, thereby indicating the efficacy of FMT in melanoma treatment (62). However, the efficacy of FMT is not stable. Antibiotics are commonly used as infection control agents in clinical settings, and their frequent use is extremely detrimental to patient prognosis; the timing of antibiotic administration during PD-1 therapy is strongly associated with patient survival (63, 64). Furthermore, interest in microbiota-modulating pharmacological agents has increased. Studies have demonstrated that treatment of the gut microbiota in melanoma-bearing mice with *Ganoderma lucidum* polysaccharides inhibits melanoma metastasis (65). *Astragalus* polysaccharides, through modulation of *Lactobacillus* spp. and *Lactobacillus johnsonii*, can enhance immune suppression within the TME, thereby promoting CD8^+^ T cell-mediated cytotoxic functions (57). Similarly, ginseng polysaccharides and inulin have been shown to augment the efficacy of PD-1 antibody therapy by modulating microbial metabolism; however, long-term follow-up and further validation of these effects are necessary.

In summary, the intricate interplay between the gut microbiota and tumor immunity highlights the potential of microbiota-targeted interventions in enhancing the melanoma treatment efficacy and overcoming immune resistance. Most current studies are in the preclinical validation stage, and clinical studies confirming the significant effects of microbiota-targeted interventions are lacking. The inconsistency of the results encourages investigation into more feasible immune mechanisms to explore therapeutic strategies that benefit most patients.

## 5 Conclusion

Rapid advancements in immunotherapy have revolutionized melanoma treatment, yet the emergence of therapeutic resistance significantly undermines the treatment efficacy and patient prognosis. Recent studies have shown that immunotherapy resistance is intricately linked to the biological and immunological behaviors of tumor cells. However, the mechanism by which the interaction between different metabolic pathways promotes melanoma progression still needs to be further clarified, as a single metabolic pathway may reduce the regulatory efficacy and cause unexpected side effects. This review consolidates current insights into alterations within the three major metabolic pathways, modifications in the tumor microenvironment, and the pivotal role of the gut microbiota in the context of melanoma immunotherapy.

Although signaling pathways, such as the BRAF and MAPK pathways, have been demonstrated to be involved in regulating melanoma cell behavior, their complex regulatory roles in immunotherapy resistance require further investigation. The current treatment strategies have not been fully validated in the clinic and have certain limitations. Achieving precise regulation of tumor cells while minimizing collateral damage to normal cells may provide novel insights into the mechanisms underlying immunotherapy resistance. With advancements in metabolomics, predictive biomarkers for metabolic pathways can be further explored, and individualized treatment and comprehensive evaluation to select the best treatment plan can be achieved.
